# Biofilms—Impacts on Human Health and Its Relevance to Space Travel

**DOI:** 10.3390/microorganisms8070998

**Published:** 2020-07-03

**Authors:** Kyle S Landry, Jose M Morey, Bharat Bharat, Nora M Haney, Sandip S Panesar

**Affiliations:** 1 Liberty Biosecurity, Expeditionary and Special Programs Division, Worcester, MA 01605, USA; jose@libertybiosecurity.com; 2 Department of Psychology, University of South Florida, St. Petersburg, FL 33620, USA; bharatb@mail.usf.edu; 3 Department of Urology, Johns Hopkins University, Baltimore, MD 21218, USA; Nhaney2@jhmi.edu; 4 Department of Neurosurgery, Stanford University, Stanford, CA 94305, USA; spanesar@stanford.edu

**Keywords:** biofilms, space, infection, health

## Abstract

As the world looks towards the stars, the impacts of endogenous and exogenous microorganisms on human health during long-duration space flight are subjects of increased interest within the space community. The presence and continued growth of bacterial biofilms about spacecraft has been documented for decades; however, the impact on crew health is in its infancy. The impacts of biofilms are well known in the medical, agricultural, commercial, and industrial spaces. It less known that biofilms are undermining many facets of space travel and that their effects need to be understood and addressed for future space missions. Biofilms can damage space crew health and spoil limited food supply. Yet, at the same time, they can benefit plant systems for food growth, nutrient development, and other biological systems that are being explored for use in space travel. Various biofilm removal techniques have been studied to mitigate the hazards posed by biofilm persistence during space travel. Because the presence of biofilms can advance or hinder humanity’s space exploration efforts, an understanding of their impacts over the duration of space flights is of paramount importance.

## 1. Introduction

It is well known that space travel subjects crew members to elevated levels of radiation that are known to increase their risk of mutations and cancer [1,2,3]. While NASA can try to protect their astronauts with shielding materials on spacecraft and spacesuits, bacteria have found a way to successfully adapt to these conditions. It has been demonstrated that bacteria can genetically and physically modify their tolerances to lower earth orbit (LEO) conditions, and one of the main mechanisms for this was the formation of biofilms [4,5,6]. While subjected to microgravity, the bacterial populations within biofilms have evolved modifications to genes and gene expression that allows them to survive in hostile environments while also increasing their virulence and pathogenicity factors [7,8,9,10]. Since astronauts will be exposed to bacterial biofilms during long-term space travel, it is imperative that the space exploration community develop an understanding of biofilm formation, persistence, and the potential mitigation of their hazards. 

## 2. Bacterial Biofilm Adaptation to the Extremes of Outer Space

The physical properties and characteristics of a biofilm are responsible for their protective and persistent nature. A bacterial biofilm is generally comprised of three components: (1) extracellular polymetric substances (EPS), (2) vegetative cells, and (3) bacterial remnants [11]. The EPS of a biofilm matrix is a complex mixture of organic material that acts as a structural glue and a physical barrier to disinfectants and antibiotics [12,13]. The EPS substance is a mixture of carbohydrates, proteins, lipids, and extracellular DNA (eDNA) [12,13]. The EPS matrix and its role in the pathogenicity and infection mechanisms is understood; however, the risks and impacts associated with biofilms and space travel is in its infancy. 

Over the past decade, there has been an increased awareness of biofilms in space-related environments [14,15,16]. The formation of biofilms on surfaces and the bio-corrosion of space hardware and life-support systems are a significant concern to all space agencies, while also becoming a growing health concern on Earth [14]. Most materials found in space craft are incapable of resisting biofilm formation and require continual maintenance to prevent formation. Additionally, critical systems, such as water pipes, air ducts and life support require service to minimize harmful effects from biofilms [16]. This is especially true for the International Space Station (ISS) or any other craft designed to support human habitation for prolonged periods of time. 

The main limitation to studying space-related biofilm formation is the ability to simulate space conditions. Even with this major hurdle, researchers have been able to utilize crew time on the ISS and have developed equipment that simulates microgravity to advance the study of biofilms in space [17,18]. One such study, presented by Kim et al., demonstrated that *Pseudomonas aeruginosa* formed a denser biofilm when grown under microgravity conditions than a biofilm grown on Earth [19,20]. The researchers were also able to demonstrate that the nutrient and gas diffusion rates within a biofilm grown under microgravity significantly impacted the overall cell density of a biofilm [20]. 

The microgravity conditions associated with LEO has also been shown to increase virulence factors in both *Salmonella* spp. and *Escherichia coli* [7,21]. Virulence and pathogenicity factors are tied to a variety of physical, metabolic, and functional gene expression of pathogens [22]. For example, flagella, a feature used for movement, is a key morphological feature that is known to be affected during growth under LEO space conditions [19,20]. Along with movement, the flagella has been shown to stimulate innate immunity, needed for the formation of microcolonies, allow cellular invasion, and promote bacterial surface adhesion [23,24]. For the transcriptome, microgravity has been shown to alter the expression of genes associated with biofilm formation, toxin production and resistance, and sporulation [25]. This also raises the question: what would happen if these enhanced pathogens were transported back to Earth following a deep space mission?

The impact of LEO conditions on the phenotypical characteristics of microorganisms has and continues to be studied to further understand what impact space travel will have on the microbial population about spacecraft. Aboard the Shenzhou VIII spacecraft, a strain of *Klebsiella pneumonia* was found to have conferred enhanced antibiotic resistance during the mission [26,27]. Interestingly, mutations continued to occur after returning to Earth [26,27]. The authors demonstrated that the mutations improved at least nine virulence/pathogenicity functions of the strain—including, but not limited to, oxidation-reduction capability and biofilm formation. Schiwon and colleagues demonstrated that over 75% of the *Staphylococcus* and *Enterococcus* species studied on the ISS demonstrated antibiotic resistance [28]. The research group postulated that most of the pathogens were normal human microflora, likely originating from the crew and cargo and that the LEO microgravity environment and the constant low-dose radiation exposure promoted the mutations. The increased pathogenicity and virulence factors of the human microbiome illustrates a serious challenge for long-duration space travel. There is no pre-flight “sterilization” process for crew members and their cargo that would limit microbial contamination and mutation during space flight [29]. To further emphasize the point, it should be noted that the standard 3-week quarantine procedures for crew members were ineffective at removing and/or limiting exposure to microorganisms that were exposed to LEO conditions [30,31].

The phenomenon known as anhydrobiosis has also been associated with bacterial biofilms in space. Contrary to the belief that water is needed to sustain bacterial life, many studies have shown that upon drying, certain bacteria are able to exist in a suspended state with little metabolic activity. Upon rehydration, the organisms are reactivated [32,33]. An experiment by Billi et al. demonstrated that dried biofilms of *Chroococcidiopsis*, when compared to their multi-layer planktonic counterparts, were able to recover faster after exposure to Mars-like conditions [34]. It was speculated that the drying process protected the organisms by minimizing the impact of free radicals and other reactive species that are present in Martian environments. Similar to bacteria suspended in anhydrobiosis, bacterial spores, when comprised in a biofilm, have also been shown to survive exposure to outer space conditions. Horneck et al. demonstrated that over the span of 6 years in outer space, *Bacillus subtilis* spores survived on the bottom layer of a biofilm [35]. A protective mechanism similar to anhydrobiosis is believed to protect bacterial spores from free radicals and cosmic galactic radiation. 

## 3. Contamination Capacity

Biofilms are a pertinent risk to the health of astronauts. Multi-species biofilms can be found on foods and surfaces along with being incorporated into drinking water systems, air circulation systems, and the shuttle structural materials themselves [36]. With the high probability of many pathogens (bacterial and fungal) having increased pathogenicity and virulence factors, the impact on crew health during long-duration space travel must be addressed. Along with health implications, it is well known that biofilms discovered on the ISS are known to be corrosive to space-related materials [29]. Biofilm formation has damaged a wide range of space station components, including polyurethane coatings and structural materials found in the majority of life support systems [14]. Even with the strict threshold of 1 CFU per 100 mL for the space shuttle water systems, higher bacterial loads have been found both pre- and post-flight [16]. 

As space flight durations increase, strategies on how to grow food have become more prominent. However, there are a multitude of problems that can arise with space-based plant growth systems due to biofilms, including contamination of the food, water, and the plant growth media. These systems also have a variety of specific hardware components, electrical circuits, and tubing that are susceptible to corrosion and contamination [37,38]. While most organisms found on the ISS are non-infectious in nature, opportunistic pathogens are present [29]. This, combined with the impacts microgravity has on the virulence, pathogenicity, and antibiotic resistance of an organism is worrisome, since it has been shown that space flight compromises one’s ability to fight off infections [39]. There is a genuine concern that the combination of increased virulence of organisms, thickness of biofilms, and compromised hosts could be detrimental to long-term space flight. Furthermore, it has been shown that space conditions alter the stability of medications that would be used in-flight to treat infections [40].

## 4. Potential Benefits

Not all biofilms have negative impacts for space travel. In fact, biofilms may provide us with clues on how we as humans can tolerate space flight. For example, Rettberg et al. used a biofilm “dosimeter” to determine if adequate UV radiation was being experienced by astronauts on their space missions to produce adequate vitamin D [41,42]. The results from the biofilm-based studies indicated that the amount of vitamin D synthesis was inadequate and oral supplementation or sunlamp UV exposure on long-duration missions was recommended. These recommendations are now routinely used during space flight.

It has been proposed that biofilms formed via bacterization could be used to promote competitive ecologies within space systems [37]. Intentional bacterial seeding has also been proposed for environmental remediation and human health on Earth [43,44,45,46,47]. This idea has been proposed and studied, though it is in its infancy, for space-based applications. For example, Ichikawa et al. describes a biofilm reactor experiment used on spaceflight missions, which uses bacteria to clean up the nitrogenous byproducts produced by aquatic organisms [48]. Other applications may involve the seeding of beneficial bacteria in waste reactors, on various food production systems, and even seeding the astronaut’s intestinal tract prior, during, and after space flight. While these ideas are noteworthy, the interplay between space and bacterial colonization needs further exploration. This is especially true since the long-term effect of radiation on beneficial bacteria has not been studied. 

## 5. Combating Biofilm: Potential Methods

### 5.1. Molecular Techniques

The widespread prevalence and proliferation of biofilm across medical devices and artificial organs has made alleviating various human diseases progressively hard. In fact, most bacterial infections are correlated with a biofilm, making biofilm infection treatment a top priority for clinical researchers [49,50,51]. During the stages of early biofilm formation, the microbial community within the biofilm is generally treatable with aggressive antibiotic treatments, but current detection methods make it difficult to diagnose biofilm infections during this stage [49]. As a result, the biofilms are only detected/diagnosed once they have established a significant foothold within the patient or on/within a medical device. A mature biofilm community is much harder to eradicate and often requires the use of multiple antibiotic treatments and/or physical removal of the infected area or device [4]. 

The formation of biofilms in environments such as hospitals, out-patient clinics, and on specialized medical devise has become a subject of increased priority over the past decade [52]. A strategy that has proven successful in combating biofilm formation on surfaces is the use of bactericidal/bacteriostatic materials, such as pure and oxidized copper materials. Numerous studies have demonstrated the correlation between the increased release of copper ions and decreased cell survival rates [53,54,55]. Similar technology and applications have been studied for space application. One research group looked at the use of copper-based antimicrobial surfaces as a way to limit the risk associated with biofilms to the crew and spacecraft [56]. As previously described, the observed antimicrobial activity was directly related to contact with the material that resulted in significant log CFU reductions of pathogenic bacteria [56]. However, the increased virulence and adaptive nature of bacteria from exposure to the extremes of outer space has been shown to increase their tolerance to such antimicrobial materials [57]. A study by Perrin et al. examined the pretreatment of materials with biosurfactants, hydrogen peroxide, silica and silver coating, but found little difference between samples coated with the materials and the negative controls [58]. However, a study by Sobisch et al. showed that a bio-deterrent surface made of silver and ruthenium had minimal bacterial growth on the surface after 6 months of in-space flight [59]. The group also demonstrated that non-coated surfaces had a significantly higher bacterial load than the metal-coated surfaces. They did mention that the bacterial isolates obtained from the metal-coated surfaces were generally Gram positive and heavily resistant to at least three antibiotics [59].

When biofilms are colonized with antibiotic-resistant organisms, the ongoing threat usually requires novel treatment approaches since single antibiotic treatments are minimally effective [60,61]. The selection, administration, pharmacokinetics (PK), and pharmacodynamics (PD) of antibiotics are considered variables when treating biofilm-related infections. Antibiotics are chosen based on their sensitivity, diffusion properties, and their efficacy in less than ideal environments [50]. The PK and PD for antimicrobial agents are used to optimize dosing regimens to provide maximum impact at the location of infection; however, these values do properly predict the diffusion rates of the antibiotics into the biofilm. Commonly, antibiotics typically used against biofilms include macrolides, lincosamides, tetracyclines, rifamycins, quinolones, fusidic acid, nitroimidazole, sulfonamides and oxazolidinones [50,51]. Due to the structural EPS component of biofilms, higher concentrations of antibiotics are often required, with a combination antibiotic therapy being ideal [62]. Depending on the location of the biofilm infection, a combination of systemic and topical antibiotics is an effective treatment. For example, the treatment for biofilm infections of the lung includes inhalation and systemic delivery of antibiotics [63,64]. This two-pronged approach greatly enhances the diffusion and overall efficacy of the antibiotics administered to the patient [63,64]. 

Combination therapy of antibiotics can be further enhanced by targeting the essential functions of bacteria. Disrupting functions such as quorum sensing (QS), nucleotide signaling, and amyloid formation have been shown to improve the outcomes of patients with biofilm infections. In biofilm communities, quorum sensing is often used to coordinate gene expression, growth, and biofilm formation within the said population [65,66]. Normally, this is for survivability; however, it has been demonstrated in numerous pathogens that the up-regulation of pertinent virulence and pathogenicity factors also occurs as a result of quorum sensing [50,67,68,69]. The upregulation of these genes can be influenced by the presence of QS-inhibitors and/or anti-QS peptides [70,71]. The QS-inhibitor meta-bromo-thiolactone has been shown to greatly reduce the biofilm-forming properties of *Pseudomonas aeruginosa* in lung cells and in microfluidic devices [70]. The researchers are now looking at imbedding QS-inhibitor compounds into/onto various surfaces to inhibit the formation of biofilms [70]. A study presented by LoVetri and Madhyastha looked at the ability of competence-stimulating peptides to inhibit biofilm formation on teeth [72]. The group found that a natural QS peptide produced by *Streptococcus mutans*, was lethal to pathogenic cells and limited the formation of biofilms [72]. Naturally occurring compounds like essential oils, ginseng and garlic extracts, and trans-stillbene are all quenchers of quorum sensing compounds. Compounds like these have also been shown to improve immune clearance and antibiotic efficacy, both in vitro and in vivo, for pathogens such as *P. aeruginosa*, *S. aureus, L. monocytogenes, and S. enteritidis* [71,73,74,75,76,77,78]. 

Along with traditional molecular techniques, researchers continue to develop novel techniques to prevent and/or remove biofilm-related infections. This is especially important for delicate environments, such as those found aboard spacecraft where commonly used treatments are not ideal. One such approach is this the use of nucleotide signaling disruptor molecules. Nucleotide signaling can be disrupted by interfering with the production of a common yet important nucleotide second messenger, cyclic diguanosine monophosphate (c-di-GMP). This messenger is vital for coordinating the phenotypical changes of bacteria that are often associated with virulence and pathogenicity factors [79,80]. Various research groups have shown that modulation the c-di-GMP pathway can be achieved by inhibiting the enzyme diguanylate cyclase (DGC) [81,82,83,84,85]. It is widely known that c-di-GMP levels within a biofilm are responsible for EPS production and biofilm stability [86,87]. Bacterial cell conversion from planktonic cells to biofilms is also directly controlled by c-di-GMP levels within the environment [88,89]. 

As a result, many research groups are looking at nucleotide signaling compounds, such as c-di-GMP as alternatives to antibiotics for the treatment of biofilms. Recently, Sambanthamoorty et al. found four small molecules that limited the DGC function of *P. aeruginosa* and *Acinetobacter baumannii* [90]. When grown in the presence of these molecules, neither culture was able to form biofilms successfully [90]. Two of the four molecules were found to have no toxic effects on eukaryotic cells. The authors strongly suggested that the further study of these molecules may lead to a safe and effective supplement to antibiotic usage. Another biochemical approach to combating biofilm formation is to inhibit the expression of bacterial amyloids that assist in the development of biofilm [91,92,93,94]. Amyloid-derived structures are the ideal building component for resilient biofilm structures. In general, amyloid protein structures are resistant to denaturation conditions and have minimal susceptibility to proteases [93,95]. A recent study found that the biofilms of *Bacillus subtilis* were repressed when the formation of their amyloid-like fibers was controlled with various combinations of small molecules [91]. 

Combating biofilm adhesion directly may be another mechanism of mitigation. Amyloid structures and other proteinaceous structures also play a major role in biofilm adhesion. It has been shown that a majority of bacteria that form biofilms have homology to a specific group of *Staphylococcus aureus* surface active proteins, known as biofilm-associated proteins (Bap), that are responsible for surface adhesion [96,97,98]. The amino acid sequence and structure models have shown that a distinctive structure of β-pleated sheets is involved in maintaining the proper structural confirmation on the cell surface for adhesion properties [99,100]. The presence of Bap-like proteins have been found and identified in *Pseudomonas* spp., *Burkolderia* spp., *Escherichia coli*, and *Vibrio* spp. [101,102,103,104]. The link between Bap and Bap-like protein expression, biofilm formation, and pathogenicity has been demonstrated for a variety of clinically relevant pathogens. In *S. aureus*, for example, researchers demonstrated that Bapdeficient mutants had minimal impact on biofilm formation and persistent infections properties [99,105]. It was also noted that the expression of Bap decreased the rate of key host-intracellular adhesion properties that would ultimately hinder biofilm formation and the establishment of chronic infections [105]. Within the opportunistic pathogen, *Enterococcus faecalis*, a Bap-like protein group known as Enterococcal surface proteins (Esp), share similar functionality to the *S. aureus* Bap proteins. The Esp are a group of high molecular weight surface proteins that promote the formation of biofilms and contribute to the pathogenicity factors of *Enterococcus faecalis* [106,107]. With a better understanding of protein interactions within biofilms, researchers have looked to various proteases to aid in the removal and prevention of bacterial biofilms. Proteases have been studied for implementation in a variety of industries plagued by biofilms. Proteases were shown to be effective at removing *Pseudomonas fluorescence*, *Bacillus* spp., *Streptococcus* spp. and, *Staphylococcal* spp. [108,109,110]. 

### 5.2. Enzymatic Techniques 

At a macro perspective, biofilm structures are comprised of various organic materials that, in theory, can be susceptible to some form of enzymatic degradation. Enzymes isolated from ubiquitous organisms to novel extremophiles possess properties that may make them effective against biofilms [111,112]. Many researchers have looked to enzymes for the removal of clinically relevant biofilms. One such approach was to use nucleases. It was found that extracellular DNA (eDNA) is an important component of the EPS matrix and aids in the adhesion and development of biofilms [113,114,115]. The eDNA is released upon cell lysis and binds with other biopolymers increasing the structural integrity of the biofilm [114]. This is especially true with amyloid structures. A study published in 2015 demonstrated how the amyloid fimbria of *Staphylococcus aureus* tightly bind to eDNA, limiting its susceptibility to nucleases [116]. The presence of eDNA not only improves fimbria adhesion properties but is also required for amyloid-fimbria expression, production, and assembly [117]. Outside of the eDNA influence on gene expression and enzymatic protection factors, eDNA also increases the interaction between bacteria and the surface via Van der Waals and acid-base interactions [12,118]. Multiple studies have shown that biofilms requiring eDNA, including *Listeria monocytogenes*, *Candida albicans*, *Streptococcus pneumoniae*, and *Pseudomonas aeruginosa*, can be greatly reduced or completely dissolved when treated with nucleases [113,119,120,121,122,123,124,125,126]. 

Proteases are another class of enzymes that have shown great promise at degrading a variety of bacterial EPS matrices. Commercially available proteases, such as Savinase and Everlase, were able to significantly degrade *Pseudomonas fluorescens* EPS attached to glass wool fibers [108]. The same group also notice that another commercial protease was not effective against the same EPS matrix and concluded that the specificity of the non-effective protease was not compatible with the protein structures within the biofilm. Krillase, a commercially available mixture of digestive proteases extracted from the Antarctic krill shrimp, has been shown to be effective at preventing and removing *Streptococcus mutans*, *Streptococcus sanguis*, *Actinomyces naeslundii*, and, *Candida albicans* biofilms [127]. The group found that 50% of bacterial density was removed with 5 min of exposure to Krillase. This was further validated via scanning electron microscopy (SEM). It is known that bacteria within biofilms produce enzymes to promote, prevent, and/or disperse biofilm colonies. The recombinant production of preventative or inhibitory proteins may alleviate substrate compatibility issues, ultimately being more effective against EPS than generic proteases. This concept was reinforced by the article published by Chen et al., which showed how an extracellular protease produced by *Staphylococcus epidermidis* inhibited *Staphylococcus aureus* biofilm formation [128]. 

Overall, the use of enzymes for the removal and prevention of biofilm formation is extremely promising. The combinatory use of multiple enzymatic classes (i.e., proteases, amylases, lipases, nucleases, etc.) would allow for a multifactorial hydrolytic profile with a high probability of success. However, the temperature-activity profiles, pH sensitivity, need for co-factors, presence of enzymatic inhibitors, and enzyme–enzyme compatibility need to be assessed for each application. The use of enzymes in lower earth orbit (LOE) as an anti-biofilm treatment technique has not been extensively studied. The effects of LOE conditions on enzyme kinetics, stability, and efficacy need to be addressed. The physiochemical changes within biofilm matrices may also change in LOE or long-duration space flight. This would impact active site accessibility and enzyme diffusion properties. 

### 5.3. Physical and Chemical Techniques 

The traditional approach to removing and preventing biofilm buildup is through chemical and/or physical removal, which encompasses the use of caustic compounds, the disassembly of equipment, and the physical scrubbing of surfaces. This is not ideal for LOE or long-duration space flight. Complex equipment, limited supplies, and an increased risk in malfunction limit the feasibility and effectiveness of this approach. Therefore, implementing recent advances in surface coatings and clean-in-place (CIP) applications for space equipment is ideal. Surfaces that limit biofouling, such as Teflon, lubricant-infused surfaces, branched-polymer surface structures, and silicone nanowire-infused surfaces, have promising properties to limit bacterial adhesion and biofilm formation [129,130,131,132,133]. 

In combination with surface coatings, advances in novel chemical techniques help alleviate the issues associated with the large-scale use of caustic agents in space. Once such technique is the use of nanotechnology. Nano-scale technology, ranging from nanoemulsions to nanotubes, have been shown to inhibit bacterial growth and biofilm formation relevant to a variety of industries [134,135,136,137]. Silver and nitric-oxide (NO) nanoparticles are currently being explored as a way to mitigate biofilm formation in chronic wounds and on contact lenses [138,139]. The application of NO-releasing nanoparticles decreased viable biofilm cell numbers by >5 log CFU of *Pseudomonas aeruginosa* and *Escherichia coli* and >3 log CFU of *Staphylococcus epidermidis*, *Staphylococcus aureus*, and *Candida albicans* at ≥6 mg/mL concentration [139]. Similarly, treatment with silver nanoparticles decreased the biofilm formation of *Staphylococcus epidermidis* and *Pseudomonas aeruginosa* by 95% and 98%, respectively, when treated with 100 nM of silver nanoparticles. The group was able to visualize the decrease in biofilm formulation on brain–heart infusion agar supplemented with Congo red dye with the addition of 50 nM silver nanoparticles. Silver nanoparticles enhanced with *Allophylus cobbe* extract significantly enhanced the antibiofilm properties of the nanoparticles along with increasing the antibiotic sensitivity of the test strains [140]. 

The use of naturally derived plant-based extracts for antimicrobial and antibiofilm activity is steadily gaining traction in a variety of industries, not just due to their efficacious properties but also due to their less aggressive nature on sensitive surfaces and equipment when compared to sodium hydroxide, sodium hypochlorite, hydrogen peroxide, and other typical CIP treatments. A recent research article tested extracts from three species of sea grass (*Enhalus acoroides, Halophila ovalis, and Halodule pinifolia*) for their ability to prevent and remove clinically relevant biofilms [141]. All three leaf extracts showed a significant biofilm reduction capability against *Escherichia coli* and *Candida albicans*. Biosurfactants, catechol(s), and essentials oils extracted from bacterial cultures, seaworms, and plant matter have all shown promising activity against biofilms [142,143,144,145]. 

In regard to long-duration space travel, replenishing disinfecting and cleaning supplies will be incredibly challenging. The ability to extract antimicrobial and antibiofilm compounds from plants or animals that can be grown and harvested during space travel is a more logical approach. These crops could also be used as a food source as well. 

## 6. Conclusions

As humans travel further and longer into outer space, we will be adapting at the same time as bacteria and fungi. Are we adapting to withstand these changes or are we becoming more susceptible? The panspermia hypothesis suggests that life not only exists across the universe but bacterial contamination occurs via propagation from planets and planetary matter. Vessels of transport include meteors, space dust, comets, planets, and spaceships themselves. While still a hypothesis, it is difficult to predict how outer space’s own bacterial contamination may interact with our protective material and food/water supplies and what the biofilm we bring from earth could mean for these extraterrestrial lifeforms and celestial bodies. Other unknowns include how our human microbiome changes in space and what that can do to our immune system and state of health. Overall, the handling and utilization of bacterial biofilm is an unavoidable challenge to promoting sustainable long-term space flight, but the potential advantages that will likely come from such research are also similarly endless.

It is essential to treat biofilms by combining a variety of methods. A constant multivariate attack strategy can lead to better and more efficient outcomes. While the clinical methods mentioned work very well on Earth, it will be interesting to observe how they unfold when biofilms are treated in the presence of microgravity. Whether the microgravity works in the favor of biofilm infection treatment is another future avenue of research to help astronauts remain healthy during longer spaceflight missions.
